# Proteotoxic Stress and Cell Death in Cancer Cells

**DOI:** 10.3390/cancers12092385

**Published:** 2020-08-23

**Authors:** Claudio Brancolini, Luca Iuliano

**Affiliations:** Department of Medicine, Università degli Studi di Udine, p.le Kolbe 4—33100 Udine, Italy; iuliano.luca@spes.uniud.it

**Keywords:** UPR, NOXA, DR5, BCL2, apoptosis, necroptosis, ferroptosis, proteotoxic stress

## Abstract

To maintain proteostasis, cells must integrate information and activities that supervise protein synthesis, protein folding, conformational stability, and also protein degradation. Extrinsic and intrinsic conditions can both impact normal proteostasis, causing the appearance of proteotoxic stress. Initially, proteotoxic stress elicits adaptive responses aimed at restoring proteostasis, allowing cells to survive the stress condition. However, if the proteostasis restoration fails, a permanent and sustained proteotoxic stress can be deleterious, and cell death ensues. Many cancer cells convive with high levels of proteotoxic stress, and this condition could be exploited from a therapeutic perspective. Understanding the cell death pathways engaged by proteotoxic stress is instrumental to better hijack the proliferative fate of cancer cells.

## 1. Proteotoxic Stress: An Introduction

Proteins are key macromolecules that play fundamental roles in almost every cellular process, from gene expression to cell/tissue protection [1]. The important and relentless actions of proteins oblige cells to supervise and guarantee their correct folding and assembling. Protein homeostasis or proteostasis is the fundamental cellular effort aimed at reaching this goal. Proteostasis is governed through a complex network of regulative mechanisms and is an essential task for cell survival [2]. The vast majority of proteins need to assume a peculiar thermodynamically stable three-dimensional structure that depends on their amino acid sequence [3]. During the folding process, proteins, particularly those presenting complex domains, can often produce folded intermediates. These intermediates can expose hydrophobic amino acid residues, thus becoming more susceptible to being stacked into a misfolded condition, a circumstance that can lead to the formation of misfolded aggregates [4].

Cells use a complex network, called the proteostasis network (PN), in order to monitor protein homeostasis. PN includes molecular chaperones and proteolytic machinery. These gene families promptly cooperate to guarantee regular proteostasis. In this manner, PN coordinates protein synthesis with folding and, if necessary, it can trigger protein degradation [5,6,7]. The importance of proteostasis maintenance becomes evident in the presence of PN dysfunctions. Inefficiency in these monitoring activities is responsible for several pathologies, including neurodegenerative diseases. Frequently, these deficiencies are age-dependent, with significant social and economic costs [7,8,9,10].

Molecular chaperones supervise protein folding, a process that requires ATP hydrolysis and a high cost in terms of energy. In particular, the chaperones of the heat shock protein (HSP) family help protein folding and are fundamental when critical conditions such as heat stress, oxidative stress, or hypoxia emerge [1]. These particular proteins are defined as HSPs because their expression is dramatically upregulated when cells are exposed to high temperatures or other forms of stress. The human genome encodes for about 330 chaperones and cochaperones [11]. The most known classes of chaperones include the ATP-dependent HSP70s, HSP90s, HSP60s (also called chaperonins), and HSP100s and the ATP-independent small HSPs (sHSPs) [11]. In many cases, chaperones are assisted in their activities by regulatory proteins called cochaperones, a large protein family that includes 244 different members. Some examples of cochaperones are the HSP40s (49 proteins) as regulators of the HSP70s and the tetratricopeptide repeat proteins (TPRs; 114 proteins) as regulators of the HSP90s. In general, cochaperones assist the functions of the chaperones by providing more selectivity and specificity toward the substrate [11,12].

Chaperones function as the main players in the maintenance of proteostasis by facilitating the folding of proteins. They usually bind to the hydrophobic polypeptide segments exposed by unfolded or not-completely-folded proteins, thus avoiding their aggregation throughout the folding process [1]. HSP70s and HSP90s are the most important members of the ATP-dependent chaperones. During the folding, they work through ATP-regulated cycles of binding and release from the target protein. This process ends when proteins are finally able to obtain their correct structure [2,13]. Moreover, some proteins are incapable of folding without the presence of chaperones, and this event determines the limit of the Anfinsen dogma. An example of this type of protein is the cytoskeletal actin [14].

Chaperones can do their duties either in cooperation with the ribosome, for example, the mammalian ribosome-associated complex (RAC) and some specialized HSP70s (HSP70L1) [15,16], or, once the polypeptide is released, alone. This is the case of HSP70s, HSP90s, and the TRiC/CCT chaperonin. In particular, TRiC are complexes structured as a double-ring that encircle, for a short time, the unfolded protein in a structure similar to a cage. In this manner, TRiC allow the correct folding and avoid the formation of aggregates [2,17]. In addition, the ATP-independent sHSPs work as a support in the maintenance of the proteins in a stable state. Through this strategy, proteins will not go under aggregation processes [18].

In general, proteins can be divided into proteins that fold easily and quickly after the interaction with the upstream chaperones, like HSP70s, and proteins that require more help during the process. The first group of proteins does not need downstream chaperones; instead, the second group of proteins is not able to complete the folding correctly and needs more specialized chaperones, like HSP90s or the chaperonins, to achieve the proper structure [19]. These “difficult” proteins are usually larger than the average and comprehend multiple domains or domains, which have complex topologies of folding. For these reasons, they need a strong interaction with the chaperones and also with the cochaperones, generating an interconnected network called “chaperome” [20].

The human PN has not yet been completely characterized in all its parts. However, investigations aimed at dissecting the network of proteins interacting with HSP90s have revealed the presence of E3 ligases (enzymes involved in the last step of ubiquitin-conjugation). This finding highlights the close relationship between the folding and protein degradation processes [21]. A detailed study, which involved about 70 chaperones, cochaperones, and proteins of the quality-control compartment, has illustrated that there is a hierarchical organization within PN. This organization is centered on the interconnected chaperone systems of HSP70 and HSP90 [22]. Another study revealed that the chaperone network can be rewired after oncogenic transformation in a new network of interactions defined as “epichaperome”, which can favor cancer cell survival [23].

Importantly, the chaperone systems have evolved several mechanisms to compensate when a single chaperone fails or is disabled. This aspect is also important from a therapeutic perspective when specific inhibitors against a chaperone are evaluated [24]. For example, the inhibition of HSP90 can promote the binding of the unfolded proteins to Hsc70, the constitutively expressed HSP70 [22]. Furthermore, between members of the BAG family of cochaperones, which act as nucleotide exchange factors of HSP70, BAG2 is the only one that has a similar substrate range to Hsc70. This evidence permits us to conclude that BAG2 could be a general cofactor, which is important in the folding mechanism of Hsc70 substrate proteins. Finally, among the interactors of Hsc70, the E3 ligase CHIP (Carboxy Terminus of HSP70-Interacting Protein) has been identified, thus further confirming the correlation between the chaperones and the ubiquitin–proteasome system (UPS) [25,26].

## 2. The Protein Quality System

In order to maintain the proteostasis, eukaryotic cells have evolved several systems monitoring quality control. These systems are different from the folding/re-folding actions of chaperones and are involved in the degradation of damaged and misfolded proteins. The most important system is represented by UPS. It works in cooperation with the lysosomal system [27,28] and plays a crucial role in several cellular processes by controlling the physiological turnover of proteins [29]. The degradation of the proteins through UPS is due to the presence of a ubiquitin tag, which is conjugated through a multistep process called ubiquitylation. A ubiquitin moiety is initially covalently linked onto Lys residues of target proteins (isopeptide-bond) and, next, elongated through the use of specific Lys of the ubiquitin itself, most frequently Lys 48. The ubiquitylation requires the coordinated action of three enzymes. The E1 ubiquitin-activating enzymes, E2 ubiquitin-conjugation enzymes, and E3 ubiquitin ligase enzymes [30,31,32]. After ubiquitylation, the tagged proteins are translocated to the 26S proteasome, an ATP-dependent protease complex found in the cytosol and in the nucleus of all eukaryotic cells. The proteasome is composed of about 50 different subunits, but it is possible to define two critical subcomplexes: the 20S catalytic core and one or two 19S regulatory subunits. The 19S particles are bound to one or both ends of the 20S component [33,34,35]. The ubiquitin tag is recognized by the 19S regulatory subunits, and, here, it is recycled by the action of deubiquitinases (DUBs). Three DUBs are associated with the 19S regulatory subunits: RPN11/POH1, USP14, and UCH-L5 [36,37,38]. This process is crucial for the degradation since the presence of the ubiquitin chain would impact sterically on the translocation from the 19S regulatory subunits to the 20S catalytic core. In fact, small molecules that inhibit these DUBs trigger cell death responses, similar to inhibitors of the catalytic portion such as bortezomib, carfilzomib, and ixazomib [36,39,40,41].

The 20S catalytic core is constituted by two heptameric rings of β-subunits and two heptameric rings of α-subunits. The α-subunits have a structural role, and the β-subunits have a catalytic role instead. In particular, β1 has a caspase-like activity, β2 has a trypsin-like activity, and β5 has a chymotrypsin-like activity [42,43,44]. A recent analysis of the proteasome and its substrates, through cryoelectron microscopy, offers a new intriguing sight into this complex process [45,46,47]. Because of the necessity of substrate unfolding, the proteasome is incapable of degrading the aggregated proteins directly. Normally, once bound to the proteasome, the substrate is unfolded by the action of six ATPase subunits (Rpt1–6) [48]. Hence, an obligatory prerequisite step for UPS-dependent degradation is substrate disaggregation through the chaperones network. The second example of such cooperation is the direct interaction between the E3 ligase CHIP and HSP70. CHIP can thus ubiquitylate chaperone–client proteins. However, this modification may still be inverted by DUBs [49]. How these conflicting mechanisms are controlled will be important for a better understanding of the protein quality control machinery.

In summary, even in the presence of UPS, protein misfolding can induce the creation of insoluble aggregates, particularly under stress situations. By contrast, autophagy can directly eliminate these aggregates via lysosomal degradation [50]. The complex molecular system involved in this task is defined as the autophagy lysosomal pathway (ALP). It includes core ATG products and additional factors, with a total of about 500 components [51]. The aggregated proteins can be accumulated in ubiquitin-positive regions, where the autophagic system is recruited by chaperones in a process known as chaperone-assisted selective autophagy (CASA) [52,53,54]. In normal unstressed conditions, the soluble proteins that need to be degraded can also be eliminated by a different type of autophagy, defined as chaperone-mediated autophagy (CMA). This response first involves the action of Hsc70, which can recognize the substrate and then the lysosomal translocation, operating through the lysosomal receptor LAMP2A (lysosome-associated membrane protein 2A). CMA avoids the formation of the autophagosome [55]. Although both UPS and ALP display an important grade of specificity toward their variety of substrates, they are connected to each other. They often compensate for each other when one of these two pathways is not working properly [56,57,58,59].

## 3. Cellular Responses to the Unfolded Proteins

Despite the presence of the chaperone systems, some errors occur during folding and many stressors such as heat, heavy metal ions, oxygen radicals, and mutations can hamper the correct folding. A misfolded or unfolded protein is not functional and can elicit a pathological condition derived from its aggregation [1,60,61,62,63]. Folding maturation in the endoplasmic reticulum (ER) is a difficult task. Proteins of the secretory pathway, if unable to fold correctly, are retained in ER and then retro-translocated to the proteasome for their degradation. The process is called ER-associated degradation (ERAD) [64]. A fundamental role in ERAD is played by the cytosolic ATPase p97 (VCP/Cdc48). This ATPase is involved in delivering the ubiquitylated unfolded proteins from ER to the proteasome through ATP hydrolysis [65]. If this system is overloaded, the accumulation of incorrectly folded proteins occurs in ER, thus leading to ER dysfunctions, including an altered redox equilibrium. These conditions are responsible for the induction of ER stress [66,67,68,69,70]. In response to ER stress, cells activate UPR (unfolded protein response) [71,72]. This adaptive response is important for sustaining cell survival. To this end, UPR blocks protein translation, increases the activation of chaperones, and potentiates the ERAD pathway. Through UPR, cells avoid the accumulation of misfolded proteins and restore the physiological condition of proteostasis. As explained above, UPR allows cells to survive the stress condition [64,70,71,72,73]. UPR is governed by three sensors: PERK (protein kinase RNA-like ER kinase), IRE1 (inositol-requiring enzyme 1) and ATF6 (activating transcription factor 6) [64,74,75]. All these sensors work in parallel to decrease ER stress. PERK and IRE1 activation can decrease protein synthesis with the consequent reduction in the number of proteins that can enter ER. The activation of ATF6 can upregulate the transcription of different chaperones involved in controlling protein folding [64].

PERK is a serine/threonine kinase that has several substrates. The best characterized is the eukaryotic translation initiation factor-2 alpha (eIF2α). PERK is able to phosphorylate eIF2α at serine 51 [70,71,76], thus blocking the CAP-dependent translation and diminishing ER stress [64]. Another notable substrate is NRF2 (nuclear factor erythroid-derived 2), the master regulator of redox homeostasis [77]. PERK can phosphorylate NRF2 on Thr 80, localized within the Neh2 domain [78]. This favors the activation of NRF2 and its nuclear import. From the nucleus, NRF2 coordinates the expression of the antioxidant response by binding the antioxidant response elements (AREs) present in the regulatory regions of several genes [79]. Additional studies have revealed that FOXO transcription factors [80,81] and diacylglycerol [82,83] can also be phosphorylated by PERK in order to reduce ER stress. Other PERK-related kinases exist that can supervise different stress conditions. Protein kinase R (PKR) is involved in the antiviral response, GCN2/EIF2AK4 (eukaryotic translation initiation factor 2 alpha kinase 4) is involved in sensing amino acid pool depletion, and, finally, HRI/EIF2AK1 (eukaryotic translation initiation factor 2 alpha kinase 1) is activated by heavy metals, heat shock, and proteasome inhibition [84]. All these kinases phosphorylate eIF2α, reduce translation, and diminish proteotoxic stress. Interestingly, HRI also confers resistance to UPS inhibitors such as bortezomib [85].

BiP/GRP78, a HSP70 family member localized into ER, is a master regulator of UPR in response to ER stress. It monitors the release and activation of the three sensors PERK, IRE1, and ATF6 [86,87]. PERK, IRE1, and ATF6 are constitutive clients of Bip/GRP78. The increase in protein unfolding, by incessantly sequestering BiP/GRP78, unleashes the three sensors and activates UPR. After the disassociation from BiP/GRP78, PERK can dimerize, and this favors its autophosphorylation and activation [88]. The activated form of IRE1, after BiP/GRP78 release, has an endoribonuclease activity that can splice a 26-base intron contained in the mRNA of the X-box binding protein 1 (XBP-1) [89]. The mature XBP-1 acts as a TF that supervises the transcription of genes involved in ERAD and protein folding [90]. Finally, the dissociation of ATF6 from BiP/GRP78 permits its translocation from ER to Golgi, where it is processed. The cleaved ATF6 can enter the nucleus where it acts as a TF to transcribe genes such as GRP78 and GRP94, which augment the ER-folding potential [91]. This sophisticated adaptive response allows cells to survive stress conditions. However, if the proteostasis restoration fails, a permanent and sustained activation of UPR can be deleterious. Initially engaged to permit cell survival, UPR can switch to triggering cell death [64,74,92].

## 4. Cell Death Pathways Activated by Proteotoxic Stress

The induction of proteotoxic stress through the use of small compounds/drugs achieves a therapeutic interest, particularly from an antitumor perspective [93]. In order to better synergize the induction of proteotoxic stress with the available therapies, it is fundamental to dissect the molecular mechanisms controlling cell death in response to proteotoxic stress.

### 4.1. The Extrinsic Pathway of Caspase Activation

**Figure 1:**
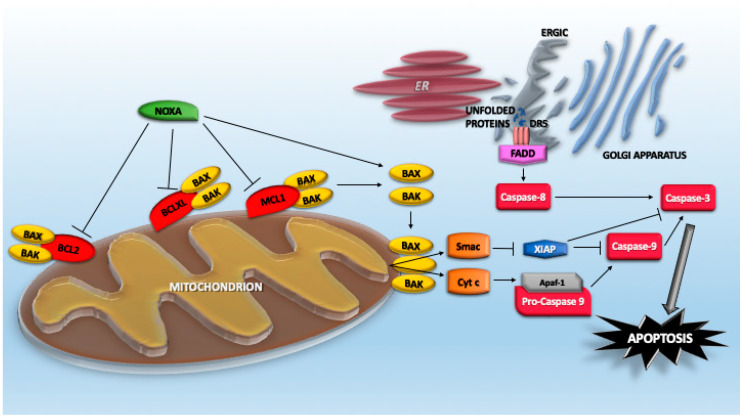
Apoptotic pathways engaged by proteotoxic stress.

It is well established that proteotoxic stress engages the mitochondrial pathway of caspase activation [94]. However, proteotoxic stress is a broad and complex pro-death insult; additionally, the extrinsic pathway is involved [95]. This role was suggested by early studies reporting the upregulation of TNFRSF10B/DR5, the TRAIL receptor, in response to ER-stressors/PERK activation, UPS inhibitors, as well as the influence of caspase-8 inhibitors on proteotoxic stress-induced cell death [39,95,96,97,98,99,100,101]. More recently, it has been proposed that UPR not only upregulates DR5 expression but misfolded proteins can directly engage with DR5 in the ER–Golgi intermediate compartment to drive the assembly of DR5 in complexes competent for caspase-8 activation (Figure 1). This activation can occur independently from the binding of its canonical extracellular ligand Apo2L/TRAIL [102]. Although the mechanism involved in such activation is unknown, a plausible hypothesis points to the release of an autoinhibitory activity that normally prevents spontaneous activation of the receptor. The increased levels of expression, the trapping in a particular membrane domain, and the priming effect of misfolded proteins could be the culprits [102,103].

In the receptor-independent activation of caspase-8 following ER stress, a contribution of RIPK1 (receptor interacting serine/threonine kinase 1) has also been proposed. The contribution appears indirect and is sustained by the use of Ripk1-deficient murine cells. The involvement of Ripk-1 in ER stressor-induced apoptosis is still mysterious. It is independent of the kinase activity from cIAP1/2 (BIRC1/2—baculoviral IAP repeat containing 1 and 2)-mediated ubiquitylation and does not involve the direct regulation of JNK/MAPK8 or CHOP [104]. ER stress can also promote inflammatory responses in the presence of chemotherapeutic regiments. Here, again, ER stress elicits TRAIL receptor upregulation, which results in a caspase-8/FADD/RIPK1-dependent activation of NF-κB. Similar to cell death, inflammatory cytokine production occurs in a ligand-independent manner. The importance of this response is testified by the protection observed in *DR5^−^*^/−^ mice from taxol-induced inflammation [103]. These studies confirm that similar to other observations, the engagement of DR5 can result in different cellular responses that are context-dependent [105].

### 4.2. The ATF Network

**Figure 2:**
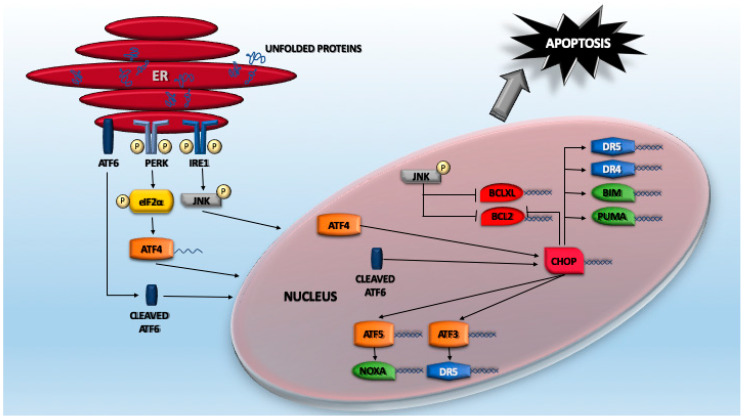
The activating transcription factor (ATF) network in response to proteotoxic stress.

A huge plethora of studies have indicated that cell death induced by proteotoxic stress can follow different routes. Certainly, the foremost investigated signaling pathway linking proteotoxic stress to apoptosis regards ER stress and the consequent UPR. A key element of this pathway is represented by ATF4 (activating transcription factor 4), a TF that belongs to the cAMP response element-binding protein (CREB)-2 family [106]. As explained above, eIF2α phosphorylation results in the attenuation of the cap-dependent protein translation, as well as the specific translation of selected mRNAs, including ATF4 itself. Normally, ATF4 protein is almost undetectable due to its very short half-life and low translation efficiency [107,108]. In fact, ATF4 levels dramatically increase in response to proteasome inhibitors because of the double effect exerted by UPR activation and the suppression of its degradation [109]. ATF4 is structured into different domains, which comprise a basic/leucine zipper domain (bZIP domain) that binds DNA. ATF4 interacts with several partners that influence its variegated transactivation activities and its stability [106,107]. As a consequence, ATF4 controls the expression of a wide range of genes that play different roles in resolving proteotoxic stress. Some of these genes are directly transcribed by ATF4, others indirectly through the action of other TFs (Figure 2). An example of a TF regulated by ATF4 is CHOP/GADD153 (CCAAT-enhancer-binding protein homologous protein), an important player of the apoptotic response [110]. Again, translation of CHOP mRNA is sustained by eIF2α phosphorylation that allows the escape from a poor translation initiation sequence [111]. Interestingly, this signaling arm is also involved in controlling ferroptosis through both GCN2-dependent and -independent mechanisms, which are elicited by cysteine depletion [112,113].

ATF4 can also trigger cell death independently from CHOP. It can promote the downregulation of the IAP family member XIAP (X-linked inhibitor of apoptosis) in a still-undefined manner. These proteins can bind and block caspase activities but can also, through a RING zinc finger domain with E3 ubiquitin ligase activity, promote ubiquitylation and the subsequent proteasomal degradation of their substrates, including caspases [114].

CHOP supervises the expression of a collection of genes. Interestingly, some of these genes are shared with ATF4, thus suggesting the existence of a feed-forward mechanism to sustain proteotoxic-dependent gene expression [115]. Similarly, the control operated by ATF6 on CHOP transcription can be viewed as a cooperative mechanism to resolve proteotoxic stress [116]. A gene under the direct transcriptional control of CHOP is DR5 [103,117,118]. A CHOP-binding site is present in the 5′-flanking region (position –281 and –216 from TSS) of the DR5 gene [117]. Moreover, ATF3, another ATF/CREB family TF that facilitates apoptotic cell death, is involved in the ER stress-mediated DR5 induction in human p53-deficient colorectal cancer cells [119,120]. TRAIL-R1/DR4 is also engaged by ER stress, although with less relevance. CHOP/ATF4 can also promote DR4 upregulation, although with differences among models and cell lines and via both transcriptional and post-transcriptional mechanisms [121,122].

ATF5 is another ATF/CREB family member under CHOP/ATF4 control (Figure 2). Transcriptional upregulation occurs via the direct binding of CARE elements in the ATF5 promoter [115,123]. Similar to ATF4 and CHOP, ATF5 is preferentially translated once eIF2 is phosphorylated. Among the ATF5-dependent genes involved in apoptosis, the BH3-only protein NOXA/PMAIP1 can be found [123]. Experimental downregulation of each of these TFs (ATF3, ATF4, ATF5, and CHOP) results in the abrogation of NOXA induction in response to proteotoxic stress. Hence, they all contribute to sustaining the feed-forward loop that drives apoptosis [115,123,124].

### 4.3. The BCL2 Family Members

NOXA/PMAIP1 is a BCL-2 proapoptotic family member that plays important roles in different apoptotic responses. NOXA is the smallest of BH3-only proteins (54 residues), and its expression is dramatically upregulated after proteotoxic stress [125]. Initially identified as a TP53 target gene [126], further studies have demonstrated that its transcription can be potently upregulated by TP53-independent mechanisms under different stress conditions, including oncogenic transformation and proteotoxic stress [127,128,129,130]. NOXA depletion impairs apoptosis in response to proteotoxic stress. NOXA can act as either sensitizer and activator by virtue of its BH3 domain, which is inserted into the hydrophobic-binding groove of multidomain proapoptotic or antiapoptotic BCL2 family members. As a sensitizer, it interacts with MCL1, BCLXL, and BCL2A1 (Figure 1). In this manner, NOXA interrupts the sequestration operated by these antiapoptotic proteins against multidomain proapoptotic proteins such as BAX and BAK. As a consequence, NOXA unleashes the pro-death activities (oligomerization and channel formation) of BAX/BAK. In contrast, as an activator, NOXA directly binds and activates BAX/BAK [131,132,133,134]. Curiously, murine Noxa contains two BH3 domains (A and B, encoded by exons 2 and 3), with only the BH3 domain B conserved in humans [126].

Additional mechanisms are used by proteotoxic stress to engage the mitochondrial pathway of caspase activation. BIM/BCL2L11 and PUMA/BBC3 are other BH3-only proteins, of which upregulation was reported in several models of proteotoxic stress and, particularly, during ER stress. The ablation of these proteins influences the death response to proteotoxic stress [84,93,94]. BIM was reported as being a transcriptional target of CHOP [135]. Similarly, PUMA expression is induced through transcriptional upregulation in response to an ER stress stimulus in a variety of human cell lines [136,137]. In addition to the action on BH3-only proteins, proteotoxic stress can downregulate BCL2 at a transcriptional level through CHOP [138] (Figure 1). Moreover, JNK activation via the IRE1 pathway triggers BCL2 and BCLXL phosphorylation and their subsequent inactivation [139,140]. Among the different routes that proteotoxic stress can engage to trigger apoptosis, the regulation of BOK (BCL2 family apoptosis regulator BOK) must also be included. This proapoptotic BCL2 family member is normally expressed at low levels. In fact, it is constitutively degraded, with a short half-life of 15 min. During proteotoxic stress, E3 ligases such as gp78, which mediates BOK degradation, become saturated because of the accumulation of misfolded proteins. Hence, BOK can accumulate to favor mitochondrial outer membrane permeabilization [141]. Normally, DNAJB12 (JB12) contributes to maintain low levels of BAK and to sustain the survival of cancer cells. This chaperon is an ER-associated Hsp40 family protein that recruits Hsp70 to the ER surface in the protein quality control system [142].

### 4.4. Additional Cell Death Responses

When proteotoxic stress advances, UPS becomes clogged by the accumulation of polyubiquitylated proteins. Blocking the proteasome affects the expression of unstable signaling proteins and, therefore, signaling pathways controlling cell survival and cell death are modulated. Two important UPS targets, controlling the survival/death switch, are the inhibitors of NF-kB, IkBɑ [143], and TP53 [144]. Furthermore, elements of the apoptotic machinery, both pro- and antiapoptotic, such as NOXA, BIM, and MCL1, also accumulate in response to UPS saturation [29,145,146]. MCL1 stabilization represents the dark side in the anticancer effect engaged by UPS inhibitors. Interestingly, multiple kinase inhibitors, such as erlotinib, rapidly enhance UPS-dependent degradation of MCL1. Erlotinib upregulates NOXA expression, which, in turn, through the action of the mitochondria-associated ubiquitin ligase MARCH5 supervises MCL1 degradation [147,148]. Similar to MCL1, other pro-survival proteins such as IAPs (XIAP, cIAP1, and cIAP2 in mammals) accumulate in response to proteotoxic stress-dependent UPS saturation [149]. The activities of these proteins can be instrumental in maintaining cell survival under stress conditions. For example, AIRAP, a proteotoxic-stress gene regulated by the master TF HSF1 (heat-shock factor 1), can regulate cell survival by controlling the levels of cIAP2 [150]. The switch between cell survival/death must also imply control over IAPs. An example is the ability of tunicamycin and thapsigargin (two ER stressors) to reduce XIAP levels in a number of mammalian cell lines [114]. XIAP translation can be reduced in a PERK-mediated manner, and ATF4 promotes its degradation, a new scenario that can contribute to reducing the threshold required for caspase activation.

As indirect consequences elicited by proteotoxic stress can favor cell death, the accumulation of ROS and the alterations of calcium homeostasis must be mentioned. These cofactors can be the deleterious corollaries of the progressive impairments in the clearance capacities normally operated by UPS and autophagy. Accumulation of unfolded proteins and aggregates impact ER and mitochondrial functions, thus leading to alterations in ROS and calcium levels that, in turn, engage further signaling events leading to cell death. How these events integrate with classic apoptotic signaling is not clear. In some studies, induction of oxidative stress can be observed in the initial phases of proteotoxic stress [84,151,152]. Certainly, the augmented levels of ROS and calcium can be responsible for the induction of alternative forms of death in response to the proteotoxic stress observed in different studies [153,154]. In general, the appearance of nonapoptotic or alternative forms of cell death in response to proteotoxic stress is a less investigated item [155,156,157]. Frequently, these necrotic-like responses appear when apoptosis is defective. Interestingly, in a model of toxicity elicited by mutant Huntingtin, a new hypothesis to explain the apoptotic/necrotic switch has been proposed. If the sequestered mutant protein is soluble, cells are characterized by hyperpolarized mitochondrial membrane potential and increased levels of reactive oxygen species, and cell death occurs via apoptosis. Instead, when mutant Huntingtin is present as aggregates, where other cellular proteins can be sequestered, a collapse in mitochondrial potential, cellular quiescence, and deactivated apoptosis occur. Overall, this response curtails cellular metabolism and leads to a slow death by necrosis [158]. Clearly, this model must be verified with general inducers of proteotoxic stress, but it is an interesting hypothesis that deserves further study. Necrotic proteotoxicity can be hampered by NRF2, possibly through the formation of autophagosomes aimed at decreasing the ubiquitylated protein aggregates [159]. Finally, in the necrotic arena, a new role of NOXA cannot be excluded, since its mitochondrial targeting domain (MTD) can trigger mitochondrial fragmentation and necrosis [160].

Necroptosis is a specific form of cell death activated through the serine/threonine kinases RIPK1 and RIPK3 and the pseudokinase MLKL [161]. Compounds that trigger necroptosis can also activate UPR [162,163]. This observation suggests some links between proteotoxic stress and necroptosis. However, as similarly discussed below for ferroptosis, it is not simple to discriminate if UPR engagement is within a pro-survival effort rather than an effective contribution to the cell death process. Importantly, a study aimed at investigating the involvement of UPR in the “classical necroptosis” induced by TNF-α discovered that two commonly used PERK inhibitors, GSK2606414 and GSK2656157, are indeed potent RIPK1 inhibitors [164]. Certainly, RIPK1, in its pleiotropic activities, can also antagonize proteotoxic stress-induced cell death. Overexpression of RIPK1 enhances the induction of autophagy and confers resistance of melanoma cells to ER stress-induced cell death [165]. Finally, during UPR and ER stress induced by hypoxia, which characterize preeclampsia, the contribution of necroptosis has been excluded. Instead, pyroptosis linked to the activation of the NLRP3 inflammasome, through the activity of thioredoxin-interacting protein (TXNIP), has been proposed [166].

Ferroptosis is a specific form of iron-dependent cell death, characterized by the accumulation of lipid peroxides due to the failure of glutathione-dependent antioxidant defenses [167,168]. Few data are available about the implications of ferroptosis in the proteotoxic stress-induced cell death. It is possible that connections exist, as recently discussed [169]. In particular, if we take into account that different ferroptotic agents can also trigger UPR [170,171], the involvement of UPR, at least in the initial phase, can be viewed as a pro-survival strategy [172], as discussed above for necroptosis. On the other hand, ROS could be the link between ferroptosis and proteotoxic stress. For example, glutathione peroxidases can regulate ferroptosis through their ability to reduce hydroperoxy groups in complex lipids and to silence lipoxygenases. However, they can also play a part during the oxidative protein-folding control in ER by reacting with protein isomerase as an alternate substrate [173].

A final important point concerns the heterogenous response of cell populations to proteotoxic stress. It is well known that although exposed to the same intensity of proteotoxic stress, some cells die while others survive. Clearly, the availability of a pool of chaperones is a critical condition. Particularly, for ER stress, the ER resident chaperone BIP is a key factor during the switch from proteostasis to proteotoxicity [174]. HSF1 is the master regulator of chaperone expression in response to proteotoxic stress. Under stress conditions, HSF1 is phosphorylated and it trimerizes and binds regulative elements in chaperone genes, thus driving their transcription [175]. Recently, a model has been proposed where membrane-less organelle foci of HSF1 regulate the cell decision in terms of survival/death. In the presence of prolonged stress, the biophysical properties of HSF1 foci can undergo a change. Small, fluid condensates enlarge into indissoluble gel-like arrangements, where HSF1 is immobilized. Consequently, chaperone gene expression decreases, leading to cell death by apoptosis [176].

## 5. Proteotoxic Stress in Cancer Cells

For a detailed discussion on proteotoxic stress and cancer, we refer to previously published reviews, some of them cited thereafter. In this section, we would like to provide only a general overview of this topic.

The protein synthesis process is intrinsically prone to errors. It has been estimated that in mammalian cells, more than 30% of newly synthesized proteins are degraded by the proteasome within minutes from their translation [177]. These quickly degraded proteins are called defective ribosomal proteins (DRiPs) or rapidly degraded polypeptides (RDPs). If not removed, DRiPs can increase proteasome loading and the consequent induction of proteotoxic stress [178]. Cancer cells generally boost protein synthesis and, therefore, DRiPs accumulate more rapidly than in normal cells [93,179]. For example, cancer cells frequently overactivate the mTORC1 pathway. This pathway is required to promote elevated levels of protein synthesis, a condition that obliges cancer cells to pay tribute to the proteasome to avoid the accumulation of misfolded proteins. This dependence from the proteasome has been exploited to kill cancer cells via small compounds blocking UPS [36,180,181,182]. The fundamental role of the proteostasis in cancer cells is further underlined by the formation of immunoproteasomes as a secondary mechanism to manage the increased proteotoxic stress arising in mutated cells for RAS, PTEN, TSC1, or mTORC1 [180,183]. Environmental conditions, which are commonly exacerbated in tumors, such as hypoxia, oxidative stress, and nutrient deprivation, are additional inducers of protein misfolding and proteotoxic stress [84,94,178].

A still poorly explored aspect of proteotoxic stress is its connection with cellular metabolism [184,185]. It seems that the switch towards an oxidative metabolism rather than glycolysis renders cancer cells resistant to the UPS inhibitor bortezomib. The regulation of the mitochondrial state could represent an additional mechanism of adaptation to proteotoxic stress that could be addressed from a therapeutic perspective [186].

In addition to the amplified levels of protein synthesis and the environmental conditions, genetic alterations accumulated in cancer are other sources of proteotoxic stress. Aneuploidy, copy number variations, and point mutations are common genetic alterations in cancers that can induce proteotoxic stress [187,188,189,190,191]. Aneuploidy is also associated with many types of stresses in cancer cells, which include both metabolic and oxidative stresses [192]. In aneuploid cells, protein complex stoichiometry imbalances are important causes of protein aggregation and proteotoxic stress induction. The uncoordinated expression of a single subunit of protein complexes, encoded on excess chromosomes, leads to its aggregate state. The excess subunits are degraded, or they aggregate, with protein aggregation nearly as effective as protein degradation for lowering the levels of excess proteins [193]. In aneuploid cells, the induction of HSF1 is also, in some way, compromised. This deficit is transduced in the impaired expression of HSP90, accumulation of misfolded proteins, and the appearance of proteotoxic stress [194]. Similarly, overexpression of genes, as well as the accumulation of mutations in coding regions, can alter normal proteostasis [195]. These mutations would produce protein variants that are more prone to misfolding, degradation, and aggregation [191].

**Figure 3:**
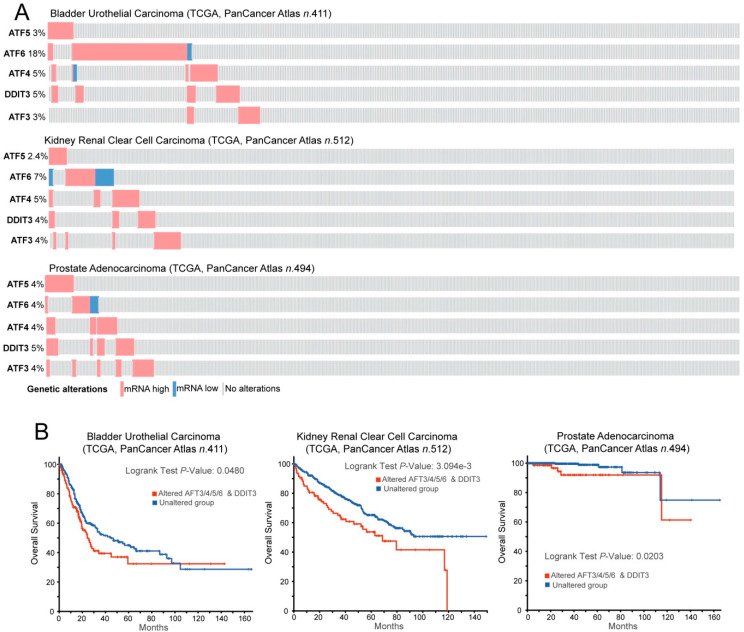
ATF factors in cancer. (**A**) Oncoprint of mRNA expression variations for the indicated TFs. Data were obtained from the TCGA database and include RNAseq data from patients, as indicated. The heatmap shows the alterations in the expression levels and was generated through cBioPortal (http://www.cbioportal.org). mRNA expression z-scores are relative to diploid samples (RNA Seq V2 RSEM). (**B**) Kaplan–Meier survival analysis related to the alterations in the mRNA levels of the ATF network. All cases were analyzed and clustered into two groups according to ATF3/4/5/6 and DDIT3/CHOP alterations in the expression levels, as illustrated in (**A**). Data were generated through cBioPortal (http://www.cbioportal.org)**.**

Cancer cells convive with proteotoxic stress by upregulating all the possible mechanisms that are able to maintain proteostasis [196,197,198,199,200,201,202,203,204]. As a consequence, cancer cells are more dependent on the presence of HSPs from UPS for their growth and survival [205,206]. Among HSPs, HSP90s and HSP70s are critical for escaping from antiproliferative signals, resisting cell death, and evading senescence. Additionally, these chaperones are involved in many distinct tracts of cancer cells, including drug resistance, angiogenesis, and metastasis [207,208]. Clearly, impacting these adaptive mechanisms has important consequences to the survival of cancer cells [209,210]. This dependence has attracted interest in developing therapeutic approaches aimed at switching-off these adaptations and thus unleashing all the dramatic consequences of the unresolved proteotoxic stress [210,211,212,213,214,215,216,217,218]. In some circumstances, adaptations to proteotoxic stress can favor the resistance to other therapeutic regiments, as observed for HSF1 and the resistance to the receptor tyrosine kinase (RTK) inhibitor lapatinib in breast cancer [219]. Interestingly, the master regulators of ER stress and UPR (ATF3/4/5/6 and CHOP) are highly expressed in a fraction of bladder, kidney, and prostate cancers, indicative of high levels of proteotoxic stress (Figure 3A). These subgroups of tumors exhibit aggressive behavior characterized by a reduction of overall survival (Figure 3B).

## 6. Conclusions

Proteostasis is a fundamental task for every cell. The evolution has sculptured elaborate interconnected mechanisms to maintain proteostasis. Some of these mechanisms have been highly conserved through evolution and, with the appearance of eukaryotic cells, each subcellular compartment has evolved a dedicated set of strategies [220,221]. Proteostasis alterations and the induction of proteotoxic stress are responsible for several pathological conditions, particularly in neurodegenerative diseases, including Huntington’s, Parkinson’s, amyotrophic lateral sclerosis, and Alzheimer’s diseases [222]. On the other hand, small compounds that are able to trigger proteotoxic stress or target the machinery resolving proteotoxic stress are actively investigated as anticancer agents [93]. Undoubtedly, the central role played by proteotoxic stress in the cell life/death decision guarantees that by studying its regulation or developing new compounds aimed to improve or impair its appearance, benefits for the human health will be generated.
